# Research trends in minimally invasive partial splenectomy: a bibliometric mapping review with cross-database comparison

**DOI:** 10.3389/fsurg.2026.1941549

**Published:** 2026-09-30

**Authors:** Wei Tian, Xu Deng, Chunyuan Yang, Jianxing Tian, Zonglong Zhu, Rui Huang, Wei Pan

**Affiliations:** Department of Hepatobiliary and Pancreatic Surgery, The People’s Hospital of Lezhi, Ziyang, China

**Keywords:** bibliometric analysis, fluorescence-guided surgery, knowledge mapping, minimally invasive partial splenectomy, robotic-assisted surgery, spleen-preserving surgery

## Abstract

**Background:**

Minimally invasive partial splenectomy (MIPS) is used for hereditary hematologic disorders, splenic cysts, hydatid disease, benign tumors, and selected traumatic lesions. However, its literature remains dispersed across patient groups, indications, and surgical specialties. This study mapped the development, contributors, knowledge base, and thematic structure of MIPS research and examined the concordance of its principal patterns across databases.

**Methods:**

Articles and reviews published from 1 January 1995 to 29 August 2026 were retrieved from the Web of Science Core Collection (WoSCC), Scopus, and PubMed. WoSCC supported the primary bibliometric and knowledge-mapping analyses; Scopus and PubMed supported cross-database comparisons. Bibliometrix, VOSviewer, and CiteSpace were used to analyze publication output, contributors, keywords, co-cited references, and citation bursts. Database overlap, annual-output correlations, and journal and theme descriptive agreement were also assessed.

**Results:**

The final datasets comprised 150 WoSCC, 154 Scopus, and 161 PubMed publications. WoSCC output increased after 2015 and reached 15 publications in 2024, the highest total among complete years. China contributed the most publications (*n* = 53), while the United States had the highest country-level total link strength. *Surgical Endoscopy*, *Journal of Pediatric Surgery*, and *Journal of Laparoendoscopic & Advanced Surgical Techniques* were the leading sources. Keyword analysis identified eight moderately coherent clusters (Q = 0.4383; mean silhouette = 0.6477), covering partial splenectomy, laparoscopic surgery, hydatid disease, hereditary spherocytosis, pediatric surgery, and operative techniques. Cross-database linkage identified 183 unique publications; 122 (66.7%) occurred in all three databases. Annual publication counts were strongly correlated for 1995–2025 (Spearman's *ρ* = 0.960–0.979) and after inclusion of partial-year 2026 data (*ρ* = 0.963–0.978; all *P* < 0.001). Eight journals appeared in all three database-specific Top-10 lists, and the leading thematic distributions were broadly similar across databases.

**Conclusion:**

MIPS research has expanded but remains relatively small, institutionally dispersed, and focused on operative feasibility, technical refinement, and perioperative outcomes. These broad patterns were consistent across the three databases, although the comparisons do not constitute independent external validation. Disease-stratified multicenter studies with standardized long-term assessment of splenic function are needed.

## 1 Introduction

The spleen is involved in immune surveillance, hematologic regulation, and clearance of senescent blood cells (1). Although total splenectomy remains necessary in some clinical settings, complete splenic removal may expose patients to long-term risks, including overwhelming post-splenectomy infection, thromboembolic events, and impaired immune function (2, 3). These concerns have led surgeons to consider spleen-preserving procedures in selected patients, particularly children and patients with benign or hereditary hematologic diseases.

Partial splenectomy was developed to remove focal splenic lesions or reduce splenic burden while retaining functional splenic tissue. With the spread of laparoscopic and robotic techniques, this procedure has gradually shifted toward minimally invasive approaches (4–9). Minimally invasive partial splenectomy (MIPS) has been reported for splenic cysts, benign splenic tumors, hereditary spherocytosis, hypersplenism, and selected traumatic lesions. Robotic assistance and indocyanine green fluorescence imaging have also been reported in a limited number of MIPS procedures, mainly to facilitate vascular dissection and intraoperative assessment of remnant perfusion (10–12). Despite these advances, the evidence remains fragmented across different diseases, age groups, institutions, and surgical specialties.

Existing reviews have summarized the indications, operative techniques, and perioperative outcomes of partial splenectomy. However, they do not show how the evidence base of MIPS has developed across pediatric surgery, hematology, minimally invasive surgery, and benign splenic disease. This distinction is clinically important because the literature spans technical feasibility, perioperative safety, disease control, and the still unresolved assessment of remnant splenic function and long-term outcomes. Bibliometric analysis offers a way to examine publication output, citation relationships, collaboration networks, and topic evolution. Visualization tools such as VOSviewer and CiteSpace can further display the structure and temporal evolution of a research field (13–15). We therefore performed a bibliometric and knowledge-mapping analysis of MIPS-related publications indexed in the Web of Science Core Collection from 1 January 1995 to 29 August 2026, with cross-database comparisons using Scopus and PubMed. This study aimed to describe the global research landscape, identify the main contributors and knowledge base, and clarify how research priorities in MIPS have changed over time.

## 2 Materials and methods

We performed a bibliometric and knowledge-mapping analysis of publications on minimally invasive partial splenectomy (MIPS). The study followed the BIBLIO reporting guideline (16), with the selection process presented in a PRISMA-style flow diagram based on PRISMA 2020 (17).

### 2.1 Data sources and search strategies

WoSCC served as the primary database for the bibliometric analysis. Scopus and PubMed were searched independently to compare literature coverage and the principal publication patterns across databases. All three databases were searched on 29 August 2026 for records published between 1 January 1995 and the search date. The starting year was set at 1995 because the earliest benchmark publication describing laparoscopic partial splenectomy was published in that year.

The search strategy was developed iteratively through pilot scoping searches, examination of the terminology used in the titles, abstracts, and author keywords of known relevant MIPS publications, and review of database-specific controlled vocabulary where applicable. Search terms were organized around two concepts: partial splenectomy and minimally invasive surgery. The procedure concept included variants of partial, subtotal, segmental, hemi-, and hemisplenectomy, whereas the surgical-approach concept included laparoscopic, robotic, robot-assisted, single-port, laparoscopy-assisted, and other minimally invasive terms. Differences in spelling, spacing, and hyphenation were incorporated into the database-specific strategies.

To assess retrieval coverage across heterogeneous terminology, the final search strategies were checked against a benchmark set of 12 known relevant publications identified through pilot searches and the reference lists of relevant reviews. The benchmark set covered different publication periods, patient populations, surgical indications, procedural terms, and minimally invasive approaches, including laparoscopic, robotic, and fluorescence-assisted partial splenectomy. Database indexing and query retrieval were assessed separately. A benchmark publication was considered successfully captured when it was indexed in the corresponding database and retrieved by the final search strategy. All benchmark publications indexed in a given database were retrieved by the corresponding search. Publications not covered by a database were labeled “not indexed” rather than “not retrieved.” The benchmark publications and database-specific retrieval results are presented in Supplementary Table S1.

WoSCC was searched using the Topic Search field, and Scopus was searched using TITLE-ABS-KEY. The PubMed strategy combined Title/Abstract terms with relevant Medical Subject Headings. The complete database-specific strategies are provided in Supplementary Table S2.

### 2.2 Eligibility and study selection

We included original articles and reviews that reported, analyzed, or substantively discussed partial or subtotal splenectomy performed through a laparoscopic, robotic, robot-assisted, laparoscopy-assisted, or other explicitly minimally invasive approach. Studies comparing open and minimally invasive surgery were eligible when the MIPS component could be identified separately.

We excluded publications that did not report actual MIPS, described only open surgery or did not specify the operative approach, mentioned MIPS only incidentally, contained a non-separable MIPS subgroup, or reported incidental splenic resection during another procedure. Ineligible publication types were also excluded.

Two investigators independently screened titles and abstracts. Full texts were consulted when eligibility could not be determined from the title, abstract, and bibliographic record. Disagreements were discussed with a third investigator.

The WoSCC search identified 293 records. Document-type restrictions excluded 23 records, and the English-language restriction excluded a further 16, leaving 254 records for content screening. No duplicate WoSCC accession numbers were found. Content screening excluded 104 records: 41 did not report actual MIPS, 14 described open surgery or had an unclear approach, 36 mentioned MIPS only tangentially or contained a non-separable subgroup, eight studies mentioned splenic resection only incidentally, and five had ineligible compound publication types. The final WoSCC dataset contained 150 publications. All analyses were rerun using this revised dataset.

The included records were exported with full bibliographic information and cited references. The extracted fields included titles, authors, affiliations, journals, publication years, abstracts, keywords, citation counts, and cited references. Figure 1 summarizes the selection process.

**Figure 1:**
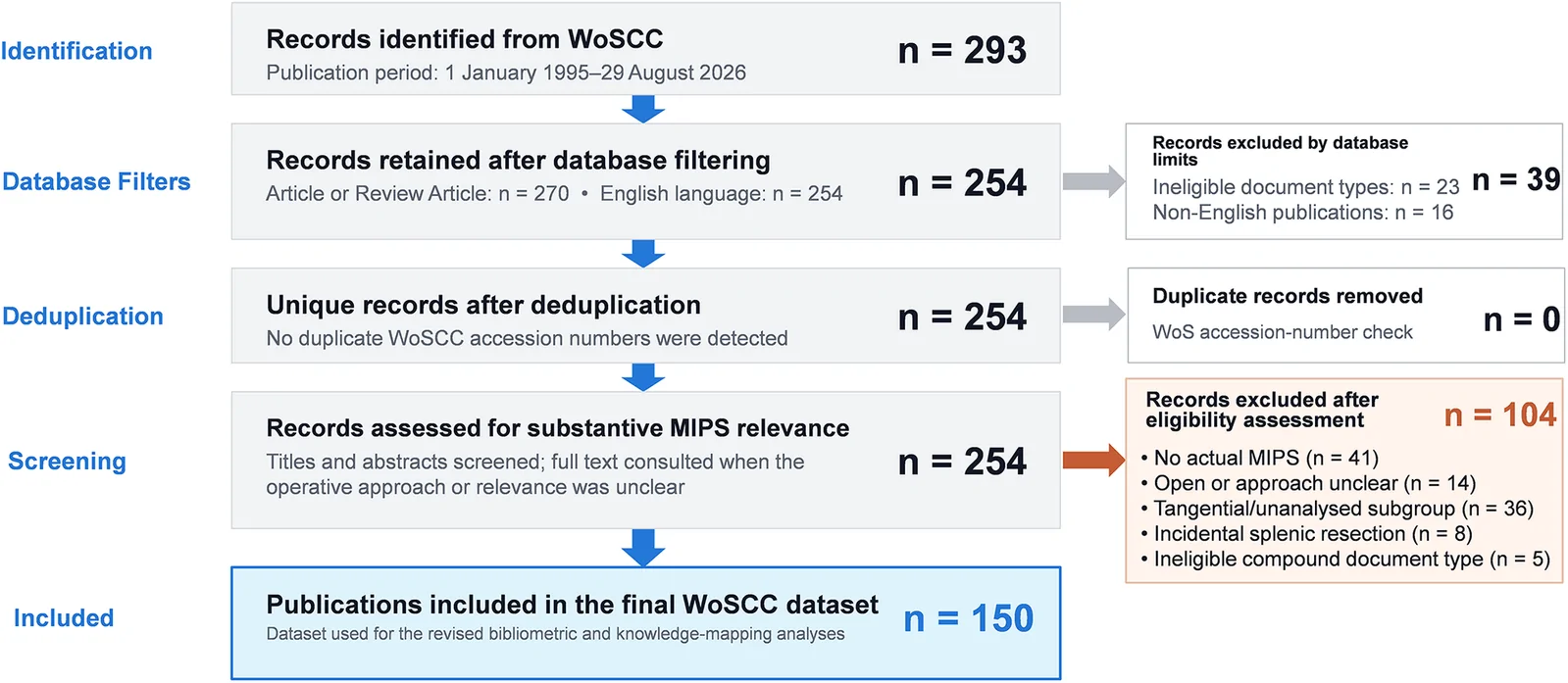
PRISMA-style flow diagram showing literature identification, database filtering, deduplication, eligibility assessment, and final inclusion from WoSCC for the bibliometric analysis of MIPS research.

### 2.3 Cross-database comparison using Scopus and PubMed

Scopus and PubMed were searched to examine the concordance of publication patterns across databases. These analyses were considered cross-database comparisons rather than independent external validation because WoSCC, Scopus, and PubMed contain overlapping publications and share common temporal trends.

The Scopus search was conducted in the TITLE-ABS-KEY field. The PubMed search combined Title/Abstract terms with relevant Medical Subject Headings, including *Laparoscopy*, *Robotic Surgical Procedures*, and *Minimally Invasive Surgical Procedures*. Both searches used the same conceptual groups for partial splenectomy and minimally invasive surgery and applied the same publication-period, language, document-type, and substantive-eligibility criteria as the WoSCC search.

The three databases were processed separately because they differ in journal coverage, indexing practices, citation metadata, and export structures. Within each database, duplicate records were checked using DOI, database-specific accession numbers—Scopus EID or PubMed PMID—and normalized title matching. Records were screened independently by two investigators, with disagreements resolved through discussion with a third investigator. Cross-database duplicates were not removed from the database-specific datasets because the datasets were not pooled. For the overlap analysis, records were linked hierarchically using exact DOI matching followed by exact normalized-title matching with publication-year and DOI-conflict checks. Records with identical titles but different DOIs were retained as separate publications unless equivalence was confirmed from the complete bibliographic information.

After database-specific eligibility screening, 154 Scopus publications and 161 PubMed publications were retained for cross-database comparison. Cross-database linkage was used to quantify literature overlap and database-specific coverage. The three database-specific datasets were then compared with respect to annual publication output, leading journal rankings, and predefined research-theme rankings.

Pairwise Spearman rank correlation coefficients were calculated for annual publication counts among WoSCC, Scopus, and PubMed. The primary analysis covered the complete publication years from 1995 to 2025, with calendar years without eligible publications retained as zero counts. Spearman correlation was selected because the annual counts were discrete and not assumed to follow a normal distribution. A sensitivity analysis was performed over 1995–2026 by additionally including the partial-year data available through 29 August 2026. All tests were two-sided and were conducted in R version 4.6.0, with *P* < 0.05 considered statistically significant. The coefficients were interpreted as measures of temporal concordance rather than independent validation because the databases contained substantially overlapping publications and shared temporal trends.

### 2.4 Data cleaning and bibliographic harmonization

Raw database exports were retained unchanged, and data cleaning was performed on working copies. Author names, institution names, country names, and author keywords were checked for obvious inconsistencies. Only one author identity required harmonization: “Uranues, S” and “Uranus, S” were standardized as “Uranüs, S” after verification based on affiliation and publication history. No semantic consolidation of all keywords was performed; synonyms, related terms, acronyms, and expanded forms were retained as provided in the source records. Because only one author-name correction was applied, the mapping is reported here rather than in a supplementary table.

### 2.5 Bibliometric analysis and visualization

Bibliometric analyses were performed using Microsoft Excel 365, the Bibliometrix R package version 5.0 in R version 4.6.0, VOSviewer version 1.6.20, and CiteSpace version 7.0.R1. Microsoft Excel and Bibliometrix were used to summarize annual publication patterns and basic bibliographic characteristics.

VOSviewer was used to construct country- and institution-level co-authorship networks, journal and cited-author co-citation networks and an all-keyword co-occurrence network. Full counting was applied, meaning that each participating author, institution, or country received one contribution from a publication. VOSviewer's association-strength method was used for network normalization.

CiteSpace was used for keyword clustering, timeline visualization, citation-burst detection, and reference co-citation analysis. The time span was set to 1995–2026, using one year per time slice; the 2026 slice included records available up to 29 August 2026. The g-index was used for within-slice node selection with k = 25. Pathfinder and Pruning Sliced Networks were applied to improve network readability. Keyword clusters were labeled using the log-likelihood ratio algorithm. Cluster quality was evaluated using modularity Q and mean silhouette values. Citation bursts were identified using CiteSpace's burst-detection algorithm, and burst strength, beginning year, and ending year were recorded.

Across the network maps, node size represented citation frequency or keyword occurrence, depending on the analysis. Links represented co-occurrence or co-citation relationships, and total link strength quantified the aggregate strength of these relationships.

## 3 Results

### 3.1 Annual publication output and citation impact

Annual output remained low until 2005, fluctuated during 2006–2014, and increased after 2015 (Figure 2). Among complete years, output peaked in 2024 (*n* = 15), followed by 2020 (*n* = 13) and 2025 (*n* = 12). By 29 August 2026, 15 publications had been identified. Mean citations per publication (MeanTCperPub) were higher but more variable in earlier years, with a peak of 155 in 2002 based on a single publication. Lower values in recent years reflect shorter citation windows.

**Figure 2:**
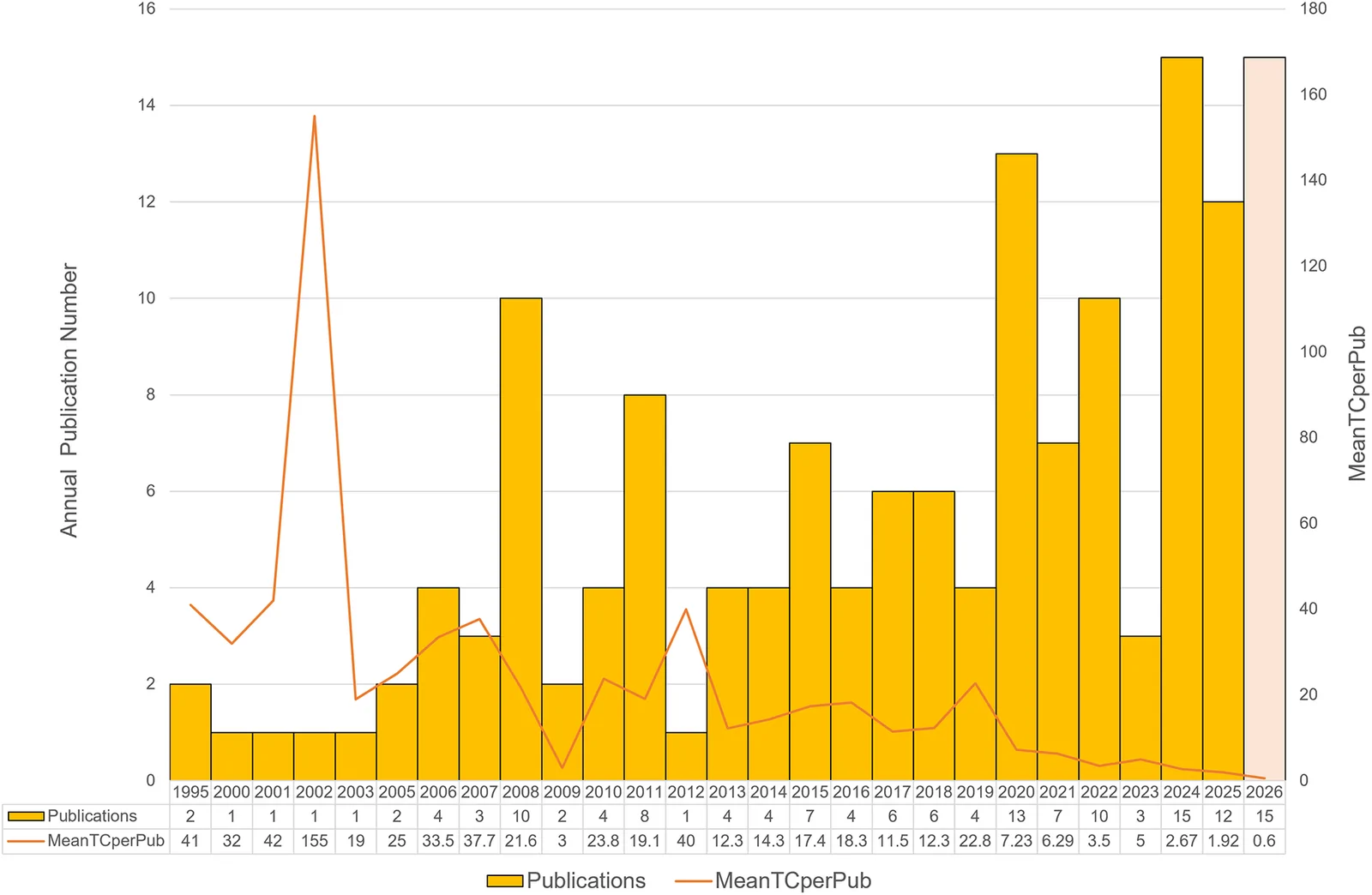
Annual publications and mean total citations per publication (MeanTCperPub) in MIPS research, 1995–2026. The lighter bar indicates partial data for 2026, updated through 29 August.

### 3.2 Distribution of countries/regions and institutions

A total of 38 countries/regions contributed to MIPS research; 21 countries/regions and 26 institutions met the minimum threshold of two publications. The 10 most productive entities in each category are presented in Table 1. China produced the most publications (*n* = 53, 35.3%), followed by the United States (*n* = 18, 12.0%) and Italy (*n* = 13, 8.7%). The United States had the highest total link strength (TLS = 6), followed by Italy and France (TLS = 5 each).

**Table 1:** Publication output and total link strength of the 10 leading countries/regions and institutions in MIPS research. A minimum threshold of two publications was applied; 21 countries/regions and 26 institutions met this threshold, and the 10 most productive entities in each category are shown.

Rank	Country	Documents	TLS	Rank	Institution	Documents	TLS
1	China	53	0	1	Fundeni Clinical Institute	7	3
2	United States	18	6	2	Sichuan University	7	0
3	Italy	13	5	3	Zhejiang University	6	0
4	France	10	5	4	Stanford University	4	5
5	Romania	7	0	5	Anhui Provincial Children's Hospital	3	0
6	Canada	6	4	6	Army Medical University	3	0
7	Germany	6	1	7	Carol Davila University of Medicine and Pharmacy	3	3
8	Japan	5	0	8	The Catholic University of Korea	3	0
9	Austria	4	3	9	Jinan University	3	0
10	Belgium	4	1	10	Medical University of Graz	3	0

TLS was derived from country- and institution-level co-authorship networks using full counting. Separate network maps were not presented because the networks were sparsely connected. TLS, total link strength.

Fundeni Clinical Institute and Sichuan University were the most productive institutions (*n* = 7 each), followed by Zhejiang University (*n* = 6) (Table 1). Stanford University had the highest institutional TLS (5), while Fundeni Clinical Institute and Carol Davila University of Medicine and Pharmacy each had a TLS of 3.

### 3.3 Publication sources and co-cited journals

*Surgical Endoscopy* published the most MIPS-related articles (*n* = 15), followed by the *Journal of Laparoendoscopic & Advanced Surgical Techniques* and the *Journal of Pediatric Surgery* (*n* = 11 each) (Figure 3A).

**Figure 3:**
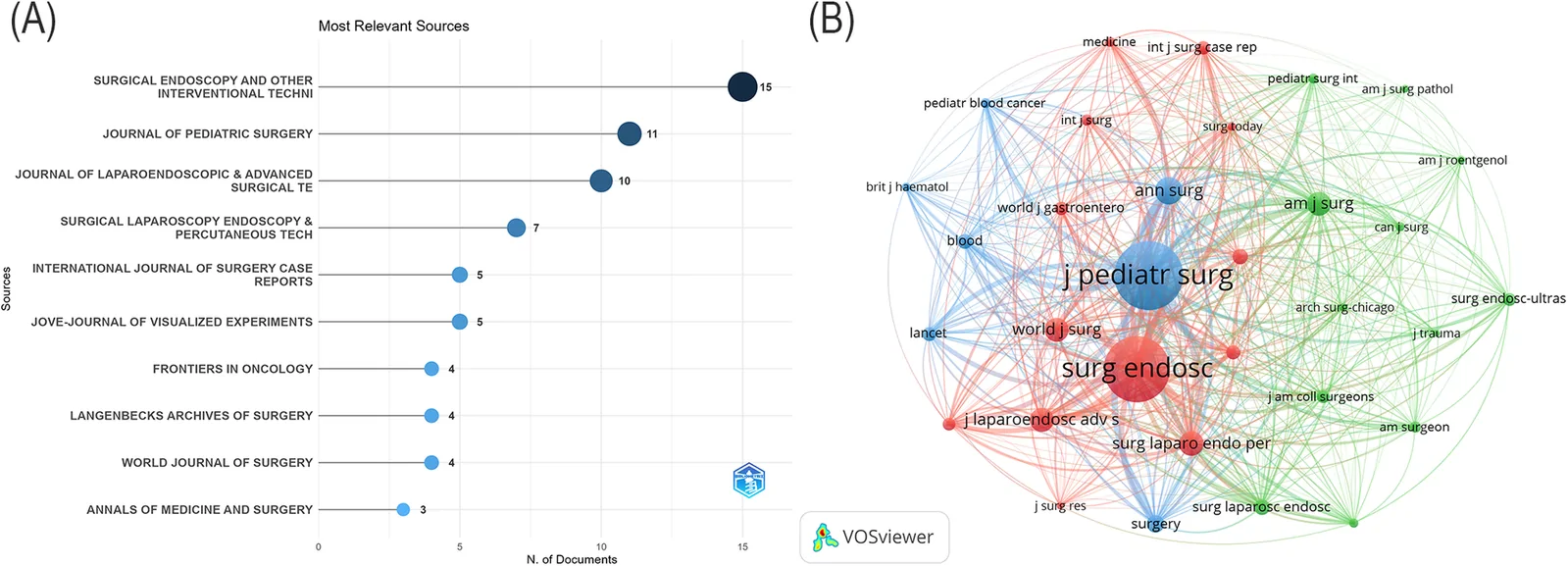
**(A)** Top 10 most productive journals. **(B)** Co-citation network of journals cited at least 20 times. The network included all 32 journals meeting the threshold. Node size represents co-citation frequency, link thickness represents total link strength, and colors denote clusters.

The co-citation network was centered on the *Journal of Pediatric Surgery* and *Surgical Endoscopy*, followed by the *Annals of Surgery* (Figure 3B). These journals connected literature from pediatric, minimally invasive, and general surgery, with additional links to hematology.

### 3.4 Author productivity and co-citation network

Vasilescu C had the longest publication span and contributed seven articles between 2006 and 2024, followed by Peng B (*n* = 6), Tudor S, and Wang X (*n* = 5 each) (Figure 4A). Recent contributions were mainly from Peng B, Wang X, Cai YQ, and Chen QJ.

**Figure 4:**
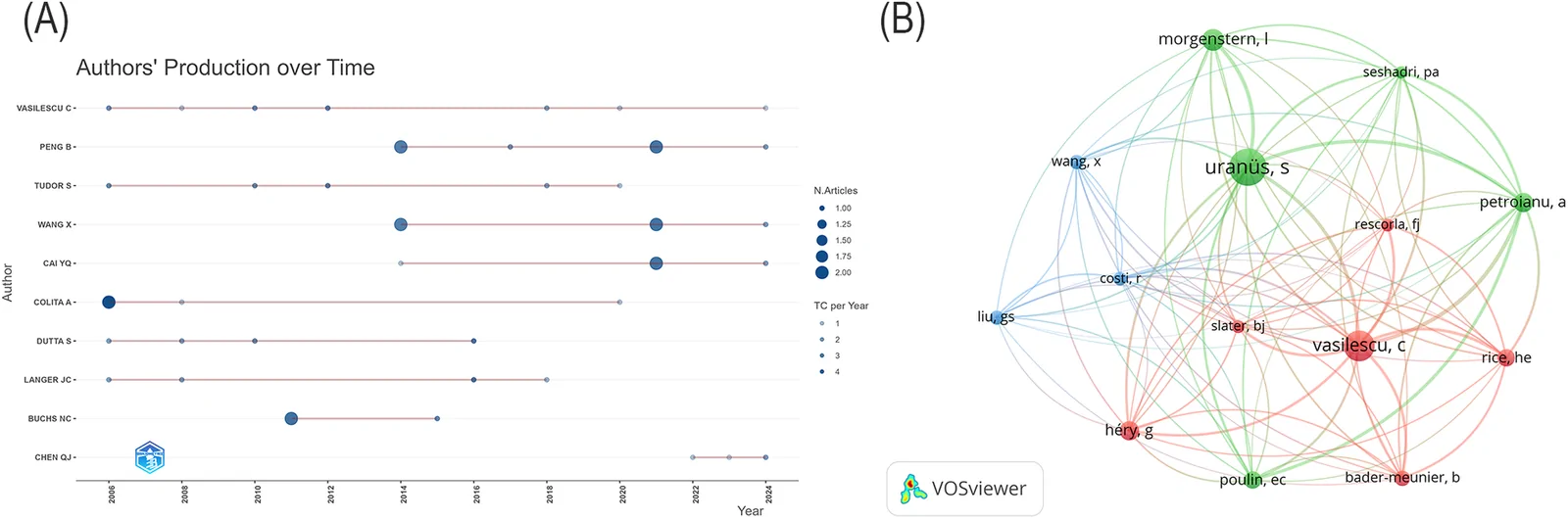
**(A)** Publication activity of the 10 most productive authors over time. Bubble size represents the number of publications, and color intensity indicates citations per year. **(B)** Co-citation network of authors cited at least 20 times. The network included all 15 cited authors meeting the threshold. Node size represents co-citation frequency, link thickness represents total link strength, and colors denote clusters.

Among authors with at least 20 co-citations, Uranüs S had the largest node, followed by Vasilescu C (Figure 4B). Morgenstern L, Petroianu A, Rice HE, Rescorla FJ, and Seshadri PA were also prominent. The three interconnected clusters reflected the shared literature on minimally invasive splenic surgery, spleen preservation, and pediatric hematology.

### 3.5 Keyword co-occurrence, clustering, and burst analysis

The keyword network was centered on partial splenectomy, management, laparoscopy, and laparoscopic partial splenectomy. These terms were closely linked to children, hereditary spherocytosis, hydatid cyst, and splenic cysts (Figure 5A).

**Figure 5:**
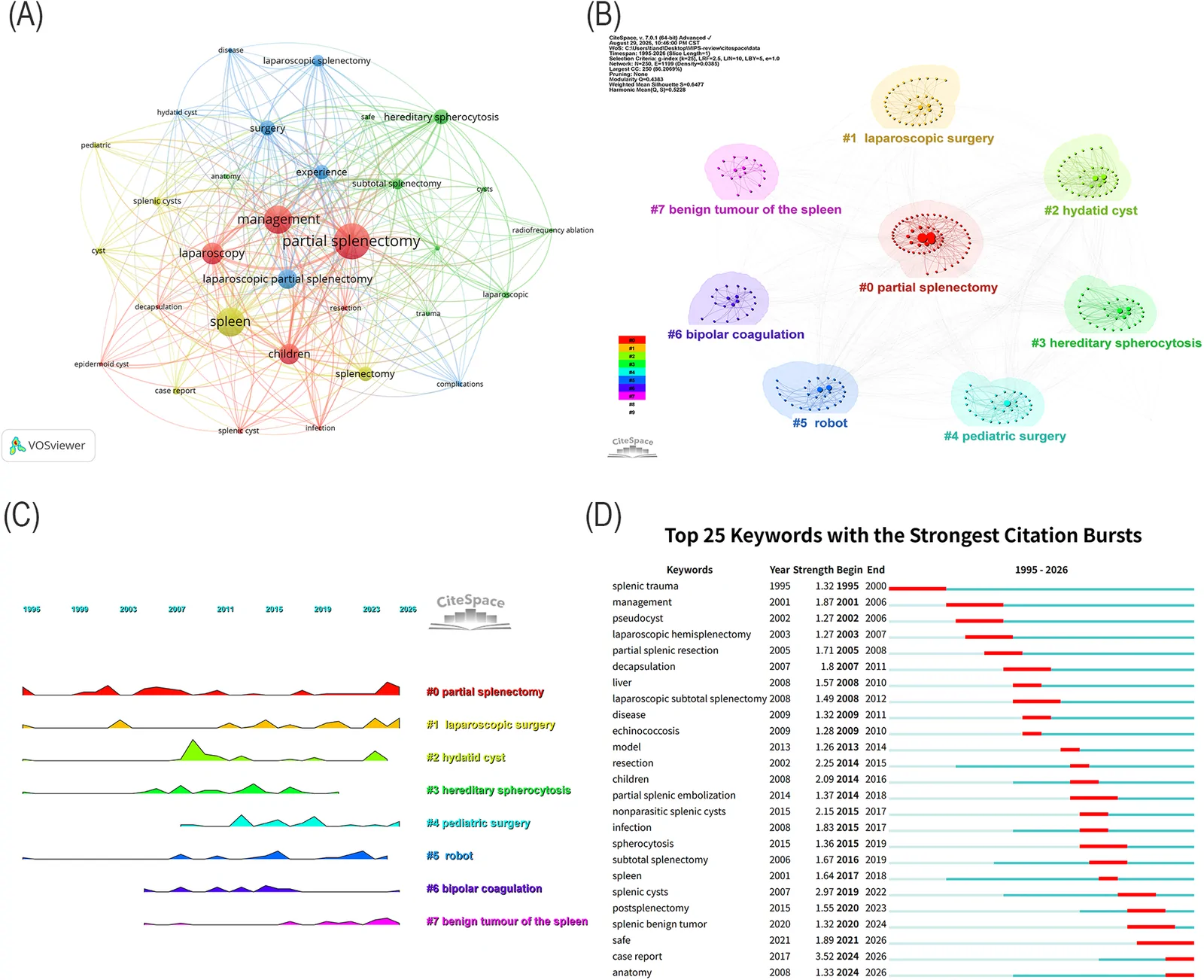
**(A)** VOSviewer co-occurrence network of all keywords occurring at least five times. The network included all 31 keywords meeting the threshold. Node size represents occurrence frequency, link thickness represents co-occurrence strength, and colors denote clusters. **(B)** CiteSpace keyword cluster map comprising eight clusters. **(C)** Landscape map showing changes in cluster activity from 1995 to 2026. **(D)** Top 25 keywords with the strongest keyword bursts from 1995 to 2026. Red segments indicate burst periods.

CiteSpace identified eight clusters with a meaningful modular structure but moderate within-cluster homogeneity (Q = 0.4383; mean silhouette = 0.6477) (Figure 5B). The clusters were therefore interpreted as broad, partially overlapping thematic groupings rather than sharply separated research domains. The largest clusters were labeled #0 partial splenectomy, #1 laparoscopic surgery, and #2 hydatid cyst. Other clusters covered hereditary spherocytosis, pediatric surgery, robot, bipolar coagulation, and benign tumor of the spleen. The landscape map showed sustained activity in partial splenectomy and more recent activity in laparoscopic surgery, pediatric surgery, benign splenic tumors, and the robot-labeled cluster (Figure 5C). However, robot did not appear among the strongest burst terms.

Early bursts included splenic trauma (1995–2000), management (2001–2006), and laparoscopic hemisplenectomy (2003–2007). Case report had the strongest burst (strength = 3.52; 2024–2026), followed by splenic cysts (2.97; 2019–2022). Safe (2021–2026), case report, and anatomy (both 2024–2026) remained active through 2026 (Figure 5D).

### 3.6 Highly co-cited references and reference co-citation analysis

Table 2 presents the 10 most frequently co-cited references in MIPS research. Héry et al. (18) ranked first with 37 co-citations, followed by Uranues et al. ((19); 31) and Poulin et al. ((20); 29). Four of the top 10 references were published in *Surgical Endoscopy*.

**Table 2:** Top 10 co-cited references in MIPS research.

Rank	Article title	First author	Year	Journal	Co-citations
1	Laparoscopic partial splenectomy: indications and results of a multicenter retrospective study (18)	Héry G	2008	Surg Endosc	37
2	Laparoscopic partial splenectomy (19)	Uranüs S	2007	Surg Endosc	31
3	Partial laparoscopic splenectomy for trauma: technique and case report (20)	Poulin EC	1995	Surg Laparosc Endosc	29
4	Long-term evaluation of the beneficial effect of subtotal splenectomy for management of hereditary spherocytosis (21)	Bader-Meunier B	2001	Blood	28
5	Feasibility and Safety of Laparoscopic Partial Splenectomy: A Systematic Review (5)	Liu GS	2019	World J Surg	26
6	Laparoscopic partial splenectomy is safe and effective in patients with focal benign splenic lesion (22)	Wang X	2014	Surg Endosc	24
7	Institutional experience with laparoscopic partial splenectomy for hereditary spherocytosis (23)	Slater BJ	2010	J Pediatr Surg	23
8	Post-splenectomy and hyposplenic states	Di Sabatino A	2011	Lancet	19
9	Laparoscopic partial splenectomy: a technical tip (24)	de la Villeon B	2015	Surg Endosc	18
10	Partial splenectomy for hereditary spherocytosis: a multi-institutional review (25)	Buesing KL	2011	J Pediatr Surg	17

The reference co-citation map showed a chronologically connected knowledge base spanning the study period (Figure 6A). Its largest connected component comprised 388 references, representing 76.98% of the original network, with 1,405 links and a density of 0.0187. Earlier influential references included Uranues et al. (19) and Héry et al. (18), whereas Liu and Fan (5), Costi et al. (9), Ouyang et al. (32), and Romboli et al. (26) were located in the more recent part of the network. The cited literature evolved from establishing the indications and rationale for spleen-preserving surgery toward evaluating laparoscopic feasibility, safety, technical refinement, and postoperative outcomes.

**Figure 6:**
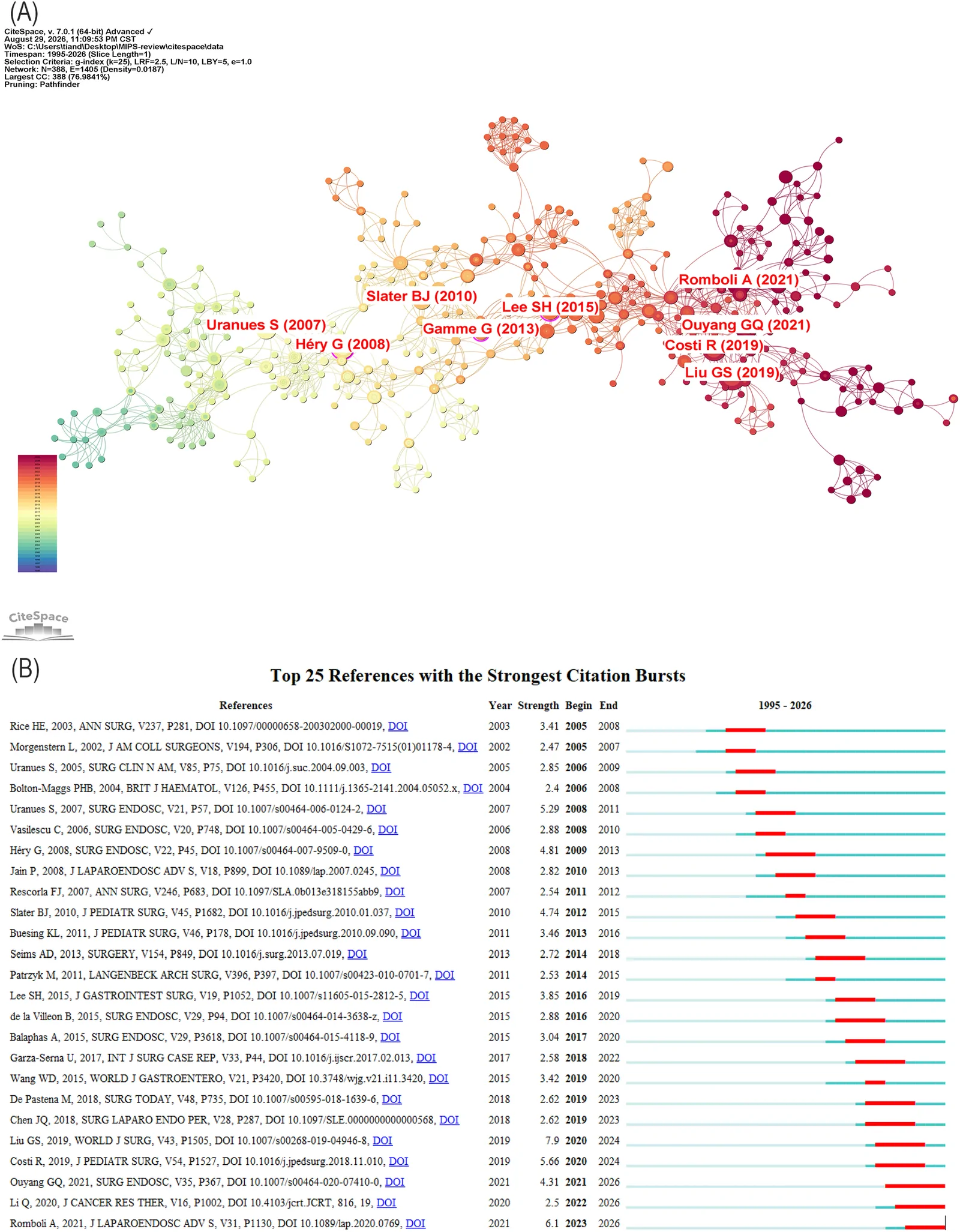
**(A)** CiteSpace reference co-citation network for 1995–2026. Each node represents a cited reference, node size reflects its co-citation frequency, and links indicate co-citation relationships. Node and link colors correspond to the year in which the co-citation connection first appeared. **(B)** Top 25 references with the strongest citation bursts. Red segments indicate the duration of each burst, while the accompanying values show burst strength and the beginning and ending years.

Citation-burst analysis identified periods during which individual references received sustained attention (Figure 6B). Liu and Fan (5) showed the strongest burst (strength = 7.90; 2020–2024), followed by Romboli et al. ((26); strength = 6.10; 2023–2026) and Costi et al. ((9); strength = 5.66; 2020–2024). Héry et al. (18) had an earlier burst from 2009 to 2013 (strength = 4.81). Three references remained in an active burst through 2026: Ouyang et al. ((32); strength = 4.31; 2021–2026), Li et al. (36; strength = 2.50; 2022–2026), and Romboli et al. ((26); strength = 6.10; 2023–2026).

### 3.7 Cross-database consistency using Scopus and PubMed

The supplementary searches retrieved 241 records from Scopus and 232 from PubMed. No within-database duplicates were identified after automated matching and manual verification. After eligibility screening, 154 Scopus records and 161 PubMed records were retained for cross-database consistency analyses (Supplementary Table S3). Cross-database linkage yielded 183 unique publications, of which 122 (66.7%) were indexed in all three databases. Only six WoSCC publications, nine Scopus publications, and eight PubMed publications were database specific, corresponding to 4.0%, 5.8%, and 5.0% of their respective datasets (Supplementary Table S4).

Annual publication output showed closely aligned temporal patterns across the three databases (Supplementary Table S5). In the primary analysis restricted to the 31 complete publication years from 1995 to 2025, annual counts were strongly correlated between WoSCC and Scopus (Spearman's *ρ* = 0.979, *P* < 0.001), WoSCC and PubMed (*ρ* = 0.960, *P* < 0.001), and Scopus and PubMed (*ρ* = 0.971, *P* < 0.001). When the partial-year data for 2026 were included in the sensitivity analysis, the corresponding coefficients were 0.978, 0.963, and 0.972, respectively (all *P* < 0.001). The inclusion of 2026 did not materially alter the magnitude or direction of the correlations. All three databases showed limited early output followed by a more marked increase in recent years.

Leading-journal patterns were broadly similar across the three databases. Surgical Endoscopy, Journal of Laparoendoscopic & Advanced Surgical Techniques, and Journal of Pediatric Surgery were the three leading journals in each database. Eight journals appeared in all three database-specific Top-10 lists. Pairwise Top-10 overlap was 80% between WoSCC and Scopus and 90% between WoSCC and PubMed and between Scopus and PubMed (Supplementary Table S6).

The predefined thematic distributions were also broadly similar. Perioperative and postoperative outcomes, spleen preservation and immunologic function, pediatric surgery, and splenic cysts were the four leading themes in all three databases. Across the 10 themes, the maximum difference in prevalence between databases ranged from 0.3 to 6.1 percentage points (Supplementary Table S7). These descriptive comparisons indicate broad concordance in temporal, journal, and thematic patterns, while reflecting substantially overlapping rather than independent literature samples.

## 4 Discussion

This study describes an expanding but still predominantly technique-oriented body of MIPS research. Publication activity increased after 2015, and the reference network linked early feasibility reports with later reviews and larger clinical series. Pediatric surgery, hereditary spherocytosis, splenic cysts, hydatid disease, and benign splenic tumors formed the main clinical settings, while the collaboration networks remained institutionally dispersed. The same broad temporal, journal, and thematic patterns were reproduced in WoSCC, Scopus, and PubMed. Unlike previous reviews that mainly summarized operative experience (5, 9, 26), this analysis shows how the literature is organized and where attention has accumulated.

### 4.1 Growth and consolidation of the evidence base

The annual pattern is consistent with the gradual consolidation of experience in a rare and technically demanding procedure. The later increase in output coincided with larger series and reviews that organized previously scattered reports around indications, operative technique, conversion, morbidity, and length of stay. Costi et al. identified 2,130 partial splenectomies, but only 371 were performed laparoscopically or robotically, and the authors cautioned that retrospective reports may overestimate favorable outcomes (9). Liu and Fan reached a similar conclusion after reviewing 252 laparoscopic procedures (5). Romboli et al. subsequently assembled 457 reported laparoscopic cases, but substantial variation in indications, methods, and outcome reporting remained (26).

The citation structure should be interpreted within that evidence base. Recent bursts were concentrated in systematic reviews and larger technical series, whereas the strongest recent keyword burst was ‘case report’; ’safe’ and ‘anatomy’ also remained active through 2026. These signals indicate continuing interest in feasibility, vascular control, and procedural safety, rather than a uniform shift toward long-term function. Publication growth alone therefore does not show that MIPS has become routine or comparatively superior. Citation patterns also favor older work, while recent articles have had less time to accumulate citations.

### 4.2 Geographic distribution and collaborative structure

China contributed more than one third of the WoSCC publications, whereas the United States and several European countries occupied more connective positions in the country network. At the institutional level, the leading centers produced only six or seven articles, and the institution with the highest link strength was not the most productive. Output and network connectivity thus captured different aspects of participation. The pattern is more consistent with distributed specialist groups than with a mature international consortium; it should not be read as a ranking of operative quality or clinical expertise.

This fragmentation may limit generalizability because partial splenectomy is sensitive to patient selection, segmental vascular anatomy, parenchymal transection, and local thresholds for conversion to total splenectomy. Outcomes from a specialist unit may not transfer directly to centers with different case mixes or operative experience. The heterogeneity reported in published reviews is compatible with this concern, although the collaboration network cannot identify its cause (5, 9, 26). A multicenter registry would be most useful if it applied common definitions for indication, approach, hemorrhage, conversion, remnant volume, recurrence, and follow-up.

### 4.3 Clinical heterogeneity and indication-specific outcomes

The journal and keyword maps placed pediatric surgery and hereditary spherocytosis alongside splenic cysts, hydatid disease, and benign tumors. These conditions share an organ-preserving operation but not a common definition of success. In hereditary hematologic disease, the main questions are durable control of anemia and hemolysis, avoidance of recurrent symptoms, and retention of clinically meaningful splenic function. Evidence from subtotal splenectomy is relevant to this rationale, although subtotal and anatomically defined partial splenectomy are not identical procedures. Long-term and multi-institutional studies have reported sustained hematologic improvement in selected patients with hereditary spherocytosis (21, 25), while disease-specific recommendations continue to emphasize careful selection (27).

Durability remains important. Hafezi et al. reported reoperation to completion splenectomy in 29% of children followed after partial splenectomy (28). For a nonparasitic cyst or benign tumor, the relevant endpoints are complete lesion treatment, preservation of a viable remnant, and freedom from recurrence; recurrence after laparoscopic cyst excision has been substantial in pediatric series (29). Hydatid disease represents another distinct stream, in which disease control and recurrence cannot be pooled with hematologic outcomes. Changes in the relative number of publications from these indications may also contribute to apparent temporal shifts in the thematic maps.

Across the three databases, perioperative and postoperative outcomes ranked ahead of spleen preservation and immunologic function. This order is consistent with a field in which operative endpoints are reported more often than direct functional assessment. A perfused remnant on imaging does not by itself establish adequate filtration or immune competence. Hematologic markers, pitted erythrocytes, scintigraphy, and immunologic measures assess different aspects of splenic activity and are not interchangeable (30, 31). Direct assessment of remnant splenic function therefore remains insufficiently represented in the literature.

### 4.4 Technical themes and emerging approaches

The most frequently co-cited references trace the expansion of laparoscopic partial splenectomy from individual technical experience to larger series. Poulin et al. reported a laparoscopic partial splenectomy after trauma (20), Uranues et al. described a broader series for localized splenic disease (19), and Héry et al. reported multicenter experience (18). Later studies examined structured dissection for focal benign lesions and temporary splenic artery occlusion to control bleeding (22, 32). This sequence is consistent with sustained attention to reproducible vascular isolation, parenchymal division, and hemostasis. Co-citation prominence does not establish that any technique is superior.

The recent activity of the robot cluster and the continued burst of ‘anatomy’ indicate emerging interest in precise segmental dissection. The signal remains secondary: robot did not appear among the strongest burst terms, while ‘case report’ and ’safe’ were more prominent. Published robotic experience includes small feasibility series and a recent pediatric comparison (12, 33); fluorescence guidance has been described in individual partial-splenectomy procedures (34). These reports justify further evaluation but do not define an established hotspot or standard of care. Comparative studies would need to account for anatomy, indication, surgeon experience, cost, and case complexity.

### 4.5 Cross-database concordance

WoSCC, Scopus, and PubMed yielded similar broad temporal patterns and identified the same leading journals and research themes. This agreement should be interpreted descriptively, as 122 of the 183 unique publications were indexed in all three databases.

Despite this substantial overlap, the comparison remains useful because it shows which findings were relatively insensitive to database selection. All three databases showed a similar increase in publication activity. Perioperative outcomes, spleen preservation, pediatric surgery, and splenic cysts were also consistently prominent across the three databases. In contrast, absolute annual counts, database-specific records, lower-ranked journals, and less frequent themes varied more readily with database coverage and indexing practices (35). These differences are particularly relevant in a small literature, where a few additional records can change the lower positions of a ranking. The cross-database analysis therefore supports the robustness of the main descriptive interpretation but should not be regarded as independent external validation.

### 4.6 Limitations and research priorities

Several limitations should be considered. Restriction to English-language publications may have underrepresented regional experience, and no single database captures every eligible surgical report. The small corpus makes annual estimates and lower-ranked contributors sensitive to individual publications and resulted in sparse country- and institution-level collaboration networks. Citation counts favor older publications, whereas the 2026 data were incomplete and recent studies had shorter citation windows. The dataset also combined pediatric and adult populations and heterogeneous indications, including hematologic diseases, splenic cysts, hydatid disease, benign tumors, hypersplenism, and trauma. Age- and indication-stratified temporal analyses were not performed because these variables were not consistently available in the bibliographic metadata and could not be assigned reliably without full-text clinical extraction. Differences in keyword indexing and theme-assignment rules may also have influenced the network and thematic results. Finally, co-citation and burst analyses reflect scholarly attention rather than methodological quality or clinical benefit.

Future studies should distinguish pediatric from adult practice and report results separately for major indications. Multicenter registries would benefit from common definitions of operative approach, hemorrhage, conversion, remnant volume, complications, recurrence, and follow-up. Long-term studies should report disease-specific control separately from remnant outcomes and include a predefined assessment of splenic function rather than relying on remnant perfusion or volume alone. This would allow technical feasibility, disease control, and durable functional preservation to be evaluated as distinct clinical outcomes.

## 5 Conclusion

MIPS research has expanded since 2015 but remains a small and institutionally dispersed field, with greater emphasis on technical feasibility, operative safety, and perioperative outcomes than on durable splenic function. Broad temporal, journal, and title-and-abstract theme patterns were similar across WoSCC, Scopus, and PubMed, although the substantial overlap among the three datasets means that this concordance should not be interpreted as independent external validation. Disease- and age-stratified multicenter studies with standardized assessment of long-term clinical outcomes and splenic function are needed.

## Data Availability

The original contributions presented in the study are included in the article/Supplementary Material, further inquiries can be directed to the corresponding author.
