# Supplementary material for: Research trends in minimally invasive partial splenectomy: a bibliometric mapping review with cross-database comparison
Source: Front Surg. 2026 Sep 30;13:1941549. doi: 10.3389/fsurg.2026.1941549 (PMC13661965; doi:10.3389/fsurg.2026.1941549)
Supplement: Supplementary file 1 [file Table1.docx]

**Supplementary Material**

**Supplementary Table S1.** Benchmark publications used to assess database-specific retrieval coverage of the final search strategies.

| **No.** | **Benchmark publication** | **Terminology or evidence represented** | **DOI** | **WoSCC** | **Scopus** | **PubMed** |
| --- | --- | --- | --- | --- | --- | --- |
| 1 | Uranüs S, Pfeifer J, Schauer C, Kronberger L, Rabl H, Ranftl G, Hauser H, Bahadori K. Laparoscopic partial splenic resection. *Surg Laparosc Endosc.* 1995;5(2):133–136. | Early terminology: partial splenic resection | Not assigned | Retrieved | Retrieved | Retrieved |
| 2 | Seshadri PA, Poulin EC, Mamazza J, Schlachta CM. Technique for laparoscopic partial splenectomy. *Surg Laparosc Endosc Percutan Tech.* 2000;10(2):106–109. | Early technical report using the standard term “partial splenectomy” | 10.1097/00019509-200004000-00013 | Retrieved | Retrieved | Retrieved |
| 3 | Smith ST, Scott DJ, Burdick JS, Rege RV, Jones DB. Laparoscopic marsupialization and hemisplenectomy for splenic cysts. *J Laparoendosc Adv Surg Tech A.* 2001;11(4):243–249. | Alternative term: hemisplenectomy; splenic cyst | 10.1089/109264201750539781 | Retrieved | Retrieved | Retrieved |
| 4 | Vasilescu C, Stanciulea O, Tudor S, Stanescu D, Colita A, Stoia R, Coriu D, Colita A, Arion C. Laparoscopic subtotal splenectomy in hereditary spherocytosis: to preserve the upper or the lower pole of the spleen? *Surg Endosc.* 2006;20(5):748–752. | Alternative term: subtotal splenectomy; hereditary spherocytosis | 10.1007/s00464-005-0429-6 | Retrieved | Retrieved | Retrieved |
| 5 | Uranues S, Grossman D, Ludwig L, Bergamaschi R. Laparoscopic partial splenectomy. *Surg Endosc.* 2007;21(1):57–60. | Standard procedural terminology | 10.1007/s00464-006-0124-2 | Retrieved | Retrieved | Retrieved |
| 6 | Héry G, Becmeur F, Méfat L, Kalfa D, Lutz P, Lutz L, Guys JM, de Lagausie P. Laparoscopic partial splenectomy: indications and results of a multicenter retrospective study. *Surg Endosc.* 2008;22(1):45–49. | Multicenter clinical evidence | 10.1007/s00464-007-9509-0 | Retrieved | Retrieved | Retrieved |
| 7 | Slater BJ, Chan FP, Davis K, Dutta S. Institutional experience with laparoscopic partial splenectomy for hereditary spherocytosis. *J Pediatr Surg.* 2010;45(8):1682–1686. | Pediatric hematologic indication | 10.1016/j.jpedsurg.2010.01.037 | Retrieved | Retrieved | Retrieved |
| 8 | Giulianotti PC, Buchs NC, Addeo P, Ayloo S, Bianco FM. Robot-assisted partial and total splenectomy. *Int J Med Robot.* 2011;7(4):482–488. | Robotic partial splenectomy; alternative minimally invasive approach | 10.1002/rcs.409 | Retrieved | Retrieved | Retrieved |
| 9 | Wang X, Wang M, Zhang H, Peng B. Laparoscopic partial splenectomy is safe and effective in patients with focal benign splenic lesion. *Surg Endosc.* 2014;28(12):3273–3278. | Focal benign splenic lesions | 10.1007/s00464-014-3600-0 | Retrieved | Retrieved | Retrieved |
| 10 | Liu G, Fan Y. Feasibility and safety of laparoscopic partial splenectomy: a systematic review. *World J Surg.* 2019;43(6):1505–1518. | Systematic-review evidence | 10.1007/s00268-019-04946-8 | Retrieved | Retrieved | Retrieved |
| 11 | Bada-Bosch I, Mata DP, de la Torre M, Ordóñez J, Blanco MD, de Agustín J. Laparoscopic partial splenectomy assisted by fluorescence in a 13-year-old girl. *Eur J Pediatr Surg Rep.* 2020;8(1):e81–e85. | Fluorescence-assisted laparoscopic partial splenectomy; pediatric application | 10.1055/s-0040-1716894 | Retrieved | Retrieved | Retrieved |
| 12 | Ouyang G, Li Y, Cai Y, Wang X, Cai H, Peng B. Laparoscopic partial splenectomy with temporary occlusion of the trunk of the splenic artery in fifty-one cases: experience at a single center. *Surg Endosc.* 2021;35(1):367–373. | Large single-center series; temporary splenic artery occlusion | 10.1007/s00464-020-07410-0 | Retrieved | Retrieved | Retrieved |

**Note:** “Retrieved” indicates that the publication was indexed in the corresponding database and retrieved by the final database-specific search strategy. “Not indexed” indicates that the publication was not covered by that database and therefore could not be retrieved. “Indexed but not retrieved” would indicate a potential omission in the search strategy. Retrieval status was assessed using the original search results before eligibility screening. DOI, digital object identifier; WoSCC, Web of Science Core Collection.

**Supplementary Table S2.** Search strategies used in WoSCC, Scopus and PubMed

| **Database** | **Search strategy** |
| --- | --- |
| **WoSCC** | TS=(("partial splenectom*" OR "subtotal splenectom*" OR hemisplenectom* OR "hemi-splenectom*" OR "partial spleen resection*" OR "partial splenic resection*" OR "segmental splenectom*" OR "segmental spleen resection*" OR "segmental splenic resection*") AND (laparoscop* OR robot* OR "minimally invasive" OR "minimal access" OR "laparoscopy-assisted" OR "laparoscopic-assisted" OR "video-assisted" OR "single-incision laparoscop*" OR "single incision laparoscop*" OR "single-port laparoscop*" OR "single port laparoscop*" OR "single-site laparoscop*" OR "single site laparoscop*" OR "laparoendoscopic single-site" OR celioscop* OR coelioscop*)) |
| **Scopus** | TITLE-ABS-KEY(("partial splenectom*" OR "subtotal splenectom*" OR hemisplenectom* OR "hemi splenectom*" OR "partial spleen resection*" OR "partial splenic resection*" OR "segmental splenectom*" OR "segmental spleen resection*" OR "segmental splenic resection*") AND (laparoscop* OR robot* OR "minimally invasive" OR "minimal access" OR "laparoscopy assisted" OR "laparoscopic assisted" OR "video assisted" OR "single incision laparoscop*" OR "single port laparoscop*" OR "single site laparoscop*" OR "laparoendoscopic single site" OR celioscop* OR coelioscop*)) |
| **PubMed** | ("partial splenectom*"[Title/Abstract] OR "subtotal splenectom*"[Title/Abstract] OR hemisplenectom*[Title/Abstract] OR "hemi splenectom*"[Title/Abstract] OR "partial spleen resection*"[Title/Abstract] OR "partial splenic resection*"[Title/Abstract] OR "segmental splenectom*"[Title/Abstract] OR "segmental spleen resection*"[Title/Abstract] OR "segmental splenic resection*"[Title/Abstract]) AND (laparoscop*[Title/Abstract] OR robot*[Title/Abstract] OR "minimally invasive"[Title/Abstract] OR "minimal access"[Title/Abstract] OR "laparoscopy assisted"[Title/Abstract] OR "laparoscopic assisted"[Title/Abstract] OR "video assisted"[Title/Abstract] OR "single incision laparoscop*"[Title/Abstract] OR "single port laparoscop*"[Title/Abstract] OR "single site laparoscop*"[Title/Abstract] OR "laparoendoscopic single site"[Title/Abstract] OR celioscop*[Title/Abstract] OR coelioscop*[Title/Abstract] OR "Laparoscopy"[MeSH Terms] OR "Robotic Surgical Procedures"[MeSH Terms] OR "Minimally Invasive Surgical Procedures"[MeSH Terms]) |

**Supplementary Table S3.** Screening outcomes and exclusion reasons for the Scopus and PubMed supplementary searches

**Panel A.** Screening and inclusion summary

| **Screening stage** | **Scopus, n** | **PubMed, n** |
| --- | --- | --- |
| Records retrieved after database filters | 241 | 232 |
| Within-database duplicate records identified and removed | 0 | 0 |
| Records remaining for eligibility screening | 241 | 232 |
| Records excluded after eligibility screening | 87 | 71 |
| **Final included records** | **154** | **161** |

**Panel B.** Reasons for exclusion after eligibility screening

| **Operational reason** | **Scopus, n** | **PubMed, n** |
| --- | --- | --- |
| No actual MIPS (e.g., total splenectomy, cyst unroofing/decapsulation or deroofing, splenopexy, ablation, or embolization) | 34 | 29 |
| Partial splenectomy was open/non-minimally invasive, or the operative approach was not identifiable | 11 | 10 |
| MIPS appeared only tangentially, as background or a recommendation, prior history, or a non-analyzed minor subgroup | 35 | 25 |
| Incidental or adjunct partial spleen resection during another primary operation | 7 | 7 |
| **Total excluded** | **87** | **71** |

**Supplementary Table S4.** Cross-database overlap and database-specific coverage of included MIPS publications.

**Panel A.** Mutually exclusive database-coverage patterns

| **Database-coverage pattern** | **Publications, n** | **Percentage of deduplicated corpus** |
| --- | --- | --- |
| Indexed in all three databases | 122 | 66.70% |
| WoSCC and Scopus only | 7 | 3.80% |
| WoSCC and PubMed only | 15 | 8.20% |
| Scopus and PubMed only | 16 | 8.70% |
| WoSCC only | 6 | 3.30% |
| Scopus only | 9 | 4.90% |
| PubMed only | 8 | 4.40% |
| **Deduplicated union** | **183** | **100.00%** |

**Panel B.** Database-specific coverage and unique contribution

| **Database** | **Included records, n** | **Shared with ≥1 other database, n** | **Database-specific records, n** | **Unique share within database** | **Coverage of deduplicated corpus** |
| --- | --- | --- | --- | --- | --- |
| WoSCC | 150 | 144 | 6 | 4.00% | 82.00% |
| Scopus | 154 | 145 | 9 | 5.80% | 84.20% |
| PubMed | 161 | 153 | 8 | 5.00% | 88.00% |

**Note:** Records were linked hierarchically using exact DOI matching followed by exact normalized-title matching with publication-year and DOI-conflict checks. Records with identical titles but different DOIs were retained as separate publications unless equivalence was confirmed from the complete bibliographic information. Percentages in Panel A were calculated using the 183-publication deduplicated union as the denominator. “Database-specific” denotes publications retrieved from only one of the three databases. MIPS, minimally invasive partial splenectomy; WoSCC, Web of Science Core Collection.

**Supplementary Table S5.** Cross-database comparison of annual MIPS publication output, 1995–2026

**Panel A.** Annual publication counts

| **Year** | **WoSCC, n** | **Scopus, n** | **PubMed, n** | **Range across databases, n** |
| --- | --- | --- | --- | --- |
| 1995 | 2 | 2 | 1 | 1 |
| 1996 | 0 | 0 | 0 | 0 |
| 1997 | 0 | 1 | 0 | 1 |
| 1998 | 0 | 0 | 0 | 0 |
| 1999 | 0 | 0 | 0 | 0 |
| 2000 | 1 | 2 | 2 | 1 |
| 2001 | 1 | 1 | 1 | 0 |
| 2002 | 1 | 2 | 2 | 1 |
| 2003 | 1 | 1 | 1 | 0 |
| 2004 | 0 | 1 | 1 | 1 |
| 2005 | 2 | 2 | 2 | 0 |
| 2006 | 4 | 4 | 4 | 0 |
| 2007 | 3 | 3 | 3 | 0 |
| 2008 | 10 | 8 | 9 | 2 |
| 2009 | 2 | 2 | 0 | 2 |
| 2010 | 4 | 4 | 5 | 1 |
| 2011 | 8 | 10 | 9 | 2 |
| 2012 | 1 | 2 | 1 | 1 |
| 2013 | 4 | 5 | 5 | 1 |
| 2014 | 4 | 4 | 4 | 0 |
| 2015 | 7 | 8 | 8 | 1 |
| 2016 | 4 | 5 | 6 | 2 |
| 2017 | 6 | 7 | 7 | 1 |
| 2018 | 6 | 7 | 8 | 2 |
| 2019 | 4 | 4 | 4 | 0 |
| 2020 | 13 | 10 | 13 | 3 |
| 2021 | 7 | 8 | 7 | 1 |
| 2022 | 10 | 12 | 10 | 2 |
| 2023 | 3 | 2 | 2 | 1 |
| 2024 | 15 | 14 | 17 | 3 |
| 2025 | 12 | 13 | 15 | 3 |
| 2026ᵃ | 15 | 10 | 14 | 5 |
| **Total** | **150** | **154** | **161** | — |

**Panel B.** Pairwise concordance in annual publication counts: primary and sensitivity analyses

| Database pair | Spearman ρ | P value | Analysis period |
| --- | --- | --- | --- |
| WoSCC–Scopus | 0.979 | <0.001 | Primary: 1995–2025 |
| WoSCC–PubMed | 0.960 | <0.001 | Primary: 1995–2025 |
| Scopus–PubMed | 0.971 | <0.001 | Primary: 1995–2025 |
| WoSCC–Scopus | 0.978 | <0.001 | Sensitivity: 1995–2026 |
| WoSCC–PubMed | 0.963 | <0.001 | Sensitivity: 1995–2026 |
| Scopus–PubMed | 0.972 | <0.001 | Sensitivity: 1995–2026 |

**Notes:** Annual counts were recalculated directly from the final database-specific included datasets. The primary correlation analysis covered the complete publication years from 1995 to 2025 and retained zero-publication years. The sensitivity analysis additionally included the partial-year counts available through 29 August 2026. Spearman coefficients indicate cross-database temporal concordance rather than independent external validation because the databases contain substantially overlapping publications. MIPS, minimally invasive partial splenectomy; WoSCC, Web of Science Core Collection.

**Supplementary Table S6.** Descriptive cross-database comparison of leading journals in MIPS research

| **Journal** | **WoSCC, n** | **Scopus, n** | **PubMed, n** | **Top-10 coverage, databases** |
| --- | --- | --- | --- | --- |
| *Surgical Endoscopy* | 15 | 16 | 17 | 3 |
| *Journal of Laparoendoscopic & Advanced Surgical Techniques* | 11 | 12 | 12 | 3 |
| *Journal of Pediatric Surgery* | 11 | 10 | 11 | 3 |
| *Surgical Laparoscopy, Endoscopy & Percutaneous Techniques* | 9 | 9 | 8 | 3 |
| *International Journal of Surgery Case Reports* | 5 | 5 | 5 | 3 |
| *Journal of Visualized Experiments* | 5 | 5 | 5 | 3 |
| *Frontiers in Oncology* | 4 | 3 | 4 | 3 |
| *World Journal of Surgery* | 4 | 4 | 4 | 3 |
| *Annals of Surgical Oncology* | 3 | 3 | 3 | 2 |
| *Langenbeck’s Archives of Surgery* | 4 | 3 | 3 | 1 |
| *Annals of Medicine & Surgery* | 3 | 1 | 3 | 2 |
| *Asian Journal of Endoscopic Surgery* | 1 | 3 | 3 | 1 |

Pairwise agreement between database-specific Top-10 lists

| **Database pair** | **Shared journals, n** | **Top-10 overlap** |
| --- | --- | --- |
| WoSCC–Scopus | 8 | 80% |
| WoSCC–PubMed | 9 | 90% |
| Scopus–PubMed | 9 | 90% |

**Note:** Database-specific Top-10 lists were generated according to publication count. When journals had the same publication count, alphabetical ordering was used for display and selection at the Top-10 cutoff. Pairwise overlap was calculated as the number of shared journals divided by 10. The comparisons were descriptive and were not interpreted as independent external validation because the databases contained substantially overlapping publications.

**Supplementary Table S7.** Cross-database consistency of major research themes in MIPS publications

| **Research theme** | **WoSCC n (%)** | **Rank** | **Scopus n (%)** | **Rank** | **PubMed n (%)** | **Rank** | **Maximum difference, percentage points** |
| --- | --- | --- | --- | --- | --- | --- | --- |
| Perioperative and postoperative outcomes | 126 (84.0%) | 1 | 120 (77.9%) | 1 | 128 (79.5%) | 1 | 6.1 |
| Spleen preservation and immunologic function | 70 (46.7%) | 2 | 67 (43.5%) | 2 | 74 (46.0%) | 2 | 3.2 |
| Pediatric surgery | 53 (35.3%) | 3 | 51 (33.1%) | 3.5 | 56 (34.8%) | 3 | 2.2 |
| Splenic cysts | 52 (34.7%) | 4 | 51 (33.1%) | 3.5 | 55 (34.2%) | 4 | 1.5 |
| Splenic tumors and vascular lesions | 35 (23.3%) | 5 | 31 (20.1%) | 5 | 33 (20.5%) | 5 | 3.2 |
| Hematologic diseases | 26 (17.3%) | 6 | 26 (16.9%) | 6 | 27 (16.8%) | 6 | 0.6 |
| Subtotal and hemi-splenectomy | 15 (10.0%) | 8 | 23 (14.9%) | 7 | 19 (11.8%) | 7 | 4.9 |
| Robotic surgery | 17 (11.3%) | 7 | 14 (9.1%) | 8 | 17 (10.6%) | 8 | 2.2 |
| Image-guided and fluorescence-assisted surgery | 11 (7.3%) | 9 | 11 (7.1%) | 9 | 12 (7.5%) | 9 | 0.3 |
| Single-incision and single-port surgery | 10 (6.7%) | 10 | 8 (5.2%) | 10 | 9 (5.6%) | 10 | 1.5 |
